# Subcutaneous, Paracardiac, and Epicardial Fat CT Density Before/After Contrast Injection: Any Correlation with CAD?

**DOI:** 10.3390/jcm10040735

**Published:** 2021-02-12

**Authors:** Caterina Beatrice Monti, Davide Capra, Alexis Malavazos, Giorgia Florini, Carlo Parietti, Simone Schiaffino, Francesco Sardanelli, Francesco Secchi

**Affiliations:** 1 Department of Biomedical Sciences for Health, Università degli Studi di Milano, 20133 Milano, Italy; caterina.monti@unimi.it (C.B.M.); davide.capra@unimi.it (D.C.); francesco.sardanelli@unimi.it (F.S.); 2 Clinical Nutrition and Cardiovascular Prevention Unit and High Specialty Center for Dietetics, Nutritional Education and Cardiometabolic Prevention, IRCCS Policlinico San Donato, San Donato Milanese, 20097 Milano, Italy; alexis.malavazos@grupposandonato.it; 3 School of Medicine and Surgery, Università degli Studi di Milano, 20122 Milano, Italy; giorgia.florini@studenti.unimi.it (G.F.); carlo.parietti@studenti.unimi.it (C.P.); 4 Department of Radiology, IRCCS Policlinico San Donato, San Donato Milanese, 20097 Milano, Italy; simone.schiaffino@grupposandonato.it

**Keywords:** coronary artery disease, adipose tissue, subcutaneous fat, beige, tomography, X-ray computed

## Abstract

Adipose tissue, in particular epicardial adipose tissue, has been identified as a potential biomarker of cardiovascular pathologies such as coronary artery disease (CAD) in the light of its metabolic activity and close anatomic and pathophysiologic relationship to the heart. Our purpose was to evaluate epicardial adipose tissue density at both unenhanced and contrast-enhanced computed tomography (CT), along with CT densities of paracardiac and subcutaneous adipose tissue, as well as the relations of such densities with CAD. We retrospectively reviewed patients who underwent cardiac CT at our institution for CAD assessment. We segmented regions of interest on epicardial, paracardiac, and subcutaneous adipose tissue on unenhanced and contrast-enhanced scans. A total of 480 patients were included, 164 of them presenting with CAD. Median epicardial adipose tissue density measured on contrast-enhanced scans (−81.5 HU; interquartile range −84.9 to −78.0) was higher than that measured on unenhanced scans (−73.4 HU; −76.9 to −69.4) (*p* < 0.001), whereas paracardiac and subcutaneous adipose tissue densities were not (*p* ≥ 0.055). Patients with or without CAD, did not show significant differences in density of epicardial, paracardiac, and subcutaneous adipose tissue either on unenhanced or contrast-enhanced scans (*p* ≥ 0.092). CAD patients may experience different phenomena (inflammation, fibrosis, increase in adipose depots) leading to rises or drops in epicardial adipose tissue density, resulting in variations that are difficult to detect.

## 1. Introduction

Epicardial adipose tissue is a metabolically active fat depot located around the heart and within the visceral wall of pericardium [1]. In particular, epicardial adipose tissue is composed of so-called “beige” adipocytes, which along with brown adipocytes are subtypes of thermogenic fats, and thus regulate metabolism and homeostasis [2]. The whitening of beige adipose tissue may be seen as a loss of its metabolic and regulatory proprieties, which can in turn lead to the onset of cardiovascular disease [3].

Epicardial adipose tissue can be assessed noninvasively via imaging studies. In particular, while transthoracic echocardiography enables one to measure epicardial adipose tissue thickness and magnetic resonance imaging allows for the calculation of its volume (and, potentially, multiparametric analysis), computed tomography (CT) permits the estimation of its volume and a density analysis based on CT numbers (obtained through reference to the water X-ray attenuation) and their translation into Hounsfield Units (HU) [4].

Due to its central role in myocardial metabolism, epicardial adipose tissue has been extensively studied with regards to its relationship with cardiac pathology and epicardial adipose tissue dysregulations, expressed as volume or density variations, have been indeed linked to coronary artery disease (CAD) [5], cardiac-related death [6], atrial fibrillation [7], and even infectious diseases [8]. In particular, Mahabadi et al. [9] observed a correlation between epicardial adipose tissue density and the occurrence of myocardial infarction, with patients presenting infarction displaying higher epicardial adipose tissue density values. Conversely, Hell et al. [10] did not identify a role for its density in predicting myocardial ischemia. Therefore, even though the association between an increased epicardial adipose tissue density and myocardial ischemia is still a matter for debate, analyzing the potential relation between its density and CAD may unravel the potential of the former as a cardiovascular risk biomarker [11]. In this regard, different studies have been conducted both with and without the intravenous administration of iodinated contrast agents [12]. As a consequence, the relationship between epicardial adipose tissue density and contrast enhancement may benefit from additional insights.

Along with epicardial adipose tissue, paracardiac adipose tissue, which is external to the parietal pericardium, has also shown a significant metabolic role and potential for beige adipocyte differentiation [3]. It may thus be hypothesized that paracardiac adipose tissue could also have a role in relation to CAD [13]. Finally, we should also consider whether subcutaneous adipose tissue, in adults mostly composed of white adipocytes, may find some correlation with CAD, albeit to a lesser extent than epicardial adipose tissue [14].

Therefore, the purpose of our study was to evaluate and compare epicardial, paracardiac, and subcutaneous adipose tissue CT density values with regards to presence of CAD in a population of patients undergoing cardiac CT for the assessment of the coronary arteries.

## 2. Experimental Section

This study was approved by our reference Ethics Committee (Ethics Committee of San Raffaele Clinical Research Hospital; protocol code “CardioRetro”, number 122/int/2017; approved on 14th September 2017 and amended 18th July 2019). Specific informed consent was waived due to the retrospective nature of this study. This study was partially supported by Ricerca Corrente funding from Italian Ministry of Health to IRCCS Policlinico San Donato.

### 2.1. Study Population

Considering the values of fat attenuation observed by Alvey et al. [15] and thus setting a potential effect size of 1 HU, with an expected proportion between CAD and non-CAD patients of 0.35, an α error of 0.05, and a power of 0.9, the sample size required to observe differences in density would be 419 patients [16]. Expecting a potential dropout due to missing or corrupted data of up to 40% we opted to retrieve between 650 and 700 patients for our study. Therefore, given the number of cardiac CTs usually performed at our institution, we estimated that we needed to go back by 42 months.

Thus, all consecutive patients who had undergone cardiac CT between January 2016 and June 2019 at our institution for coronary artery evaluation, performed under the responsibility by the same radiologist (F.Se.) to ensure homogeneity of the technical protocol, were retrospectively retrieved. Patients with missing or corrupted data, namely duplicate individuals, those who were missing either the unenhanced or contrast-enhanced scan, or those with heavily artefacted images were excluded from the analysis. We considered patients who showed at least either a moderate (between 50% and 75%) stenosis on one main coronary artery or a mild (less than 50%) stenosis on two main coronary arteries as positive for CAD.

### 2.2. Image Acquisition

All examinations were performed on a 64-row CT scanner (Somatom Definition, Siemens Healthineers, Erlangen, Germany), with retrospective electrocardiographic gating. Gantry rotation time was 0.33 s, pitch from 0.2 to 0.5. The reconstruction parameters for the angiographic phase scan were set as follows: slice thickness 1.5 mm; reconstruction interval 0.6 mm; and matrix size 512 × 512. Tube voltage was set between 100 and 120 kVp with tube current set accordingly, in relation to body size. An unenhanced scan with a slice thickness of 3.0 mm was acquired, then a bolus of contrast material of 1 mL/kg (Iopamiro 400, Bracco Imaging S.p.A., Milan, Italy) followed by saline solution in the range of 30–70 mL was intravenously injected by means of a power injector (Empower CTA, EZEM, Westbury, NY, USA) at a flow rate of 3.0–5.0 mL/s according to the patient’s venous access features. A test-bolus technique was used, and a time-attenuation curve was obtained by measuring the enhancement within a region of interest located in ascending aorta immediately above the bulb. Contrast arrival time was determined from the time-to-peak enhancement in the region of interest, and it was used to plan the scan delay for a full-bolus diagnostic scan.

### 2.3. Image Analysis

**Figure 1:**
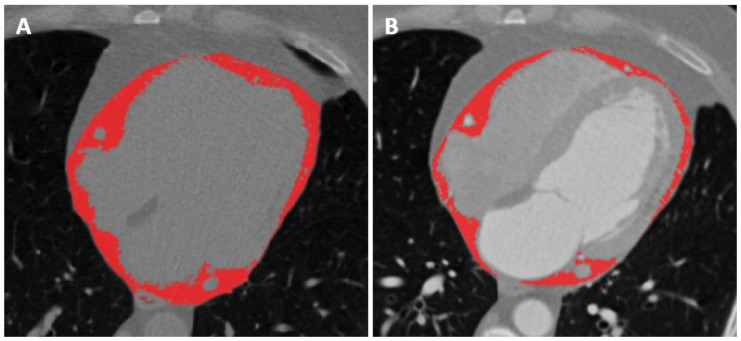
Segmentation of epicardial adipose tissue with application of thresholding, thus only including voxels with a density values between −190 and −30 HU, on both the unenhanced (**A**) and contrast-enhanced (**B**) scans. The paracardiac adipose tissue lies right outside the pericardium, surrounding the epicardial adipose tissue.

One reader with 1 year of experience in cardiac CT performed image segmentation with the supervision of another reader with 4 years of experience. Image segmentation was performed using 3D Slicer software, version 4.10.2 [17]. For each patient, the reader placed two regions of interest (ROI) of 1 cm diameter on paracardiac and subcutaneous adipose tissue, respectively. All segmentations were performed on a CT slice which permitted a clear visualization of the adipose tissue pertaining to such structures. When segmenting epicardial adipose tissue, particular attention was posed to the fact that the ROIs were not to exit the pericardium, whereas when segmenting the paracardiac adipose tissue, the ROI was not to enter the pericardium. The whole pericardium was also segmented on a representative CT slice. All segmentation files were then saved as NIfTI masks [18]. Images were subsequently cropped according to masks obtained from the ROIs, and the threshold was according to adipose tissue attenuation values, estimated between −190 and −30 HU on both enhanced and unenhanced scans [19,20]. The average attenuation values for each region were then automatically calculated and recorded. An example of epicardial adipose tissue segmentation with the application of the aforementioned threshold on unenhanced and contrast-enhanced scans is depicted in Figure 1.

**Figure 2:**
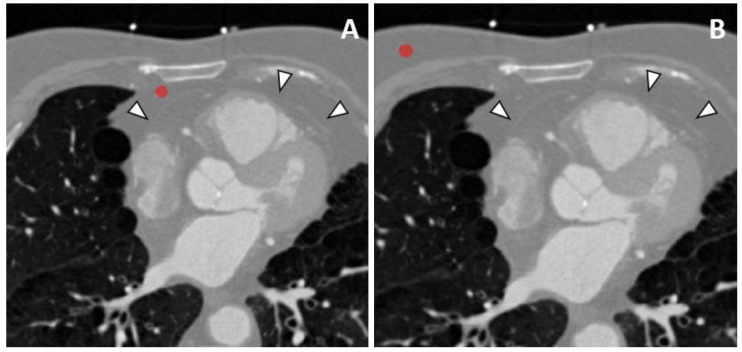
Segmentation of paracardiac (**A**) and subcutaneous (**B**) adipose tissue, by placing regions of interest with a diameter of 1 cm which will then be thresholded for fat. The pericardium may be seen as a thin, hyperdense line marked by white arrowheads separating the epicardial (inside) and paracardiac adipose tissue (outside).

Depiction of regions of interest encompassing the paracardiac and subcutaneous adipose tissue are instead reported in Figure 2. 

### 2.4. Statistical Analysis

The Shapiro–Wilk test was used to assess data distribution. Normal variables were reported as mean ± standard deviation, whereas non-normal data were reported as median and interquartile range (IQR). To appraise density differences among fat tissues, Student *t*-test or Wilcoxon test for paired data were used according to data distribution. A Kruskal–Wallis test was used to compare more than two groups, and Dunn post-hoc analyses were performed to assess differences among each pair of distributions. χ^2^ statistics was used to compare categorical variables. Statistical analysis was performed using R (v.4.0.2, The R Foundation for Statistical Computing, Vienna, Austria). *p*-values ≤ 0.05 were considered as statistically significant [21].

## 3. Results

### 3.1. Study Population

Out of 670 consecutive patients initially retrieved, 161 were excluded prior to image retrieval as 6 represented duplicate patients with different data, and 155 lacked unenhanced scans. Thus, 509 patient files were retrieved. A further 29 patients were excluded after image retrieval, as their scans presented heavy artifacts which could hinder image analysis, leading to 480 patients being ultimately included in the study. Among them, 186 (39%) were female, median age was 66 years (IQR 56–73 years). Over half of patients (299, 62%) were referred to CT for suspected CAD, being either deemed at high risk of CAD, positive at a stress test, or symptomatic, 77 (16%) underwent CT for follow-up of a known CAD, and 104 (22%) were referred for other reasons, such as pre- or post-surgical evaluation. At CT, 164 (34%) patients had CAD. Patients with CAD were older than those without (71 years (IQR 64–76 years) versus 62 years (IQR 54–70 years), *p* < 0.001). In addition, among 164 patients with CAD, we had more often males than females (patients with CAD were 71% males and 29% females, whereas those without CAD were 56% males and 44% females, *p* = 0.002).

### 3.2. Fat Density Values

**Figure 3:**
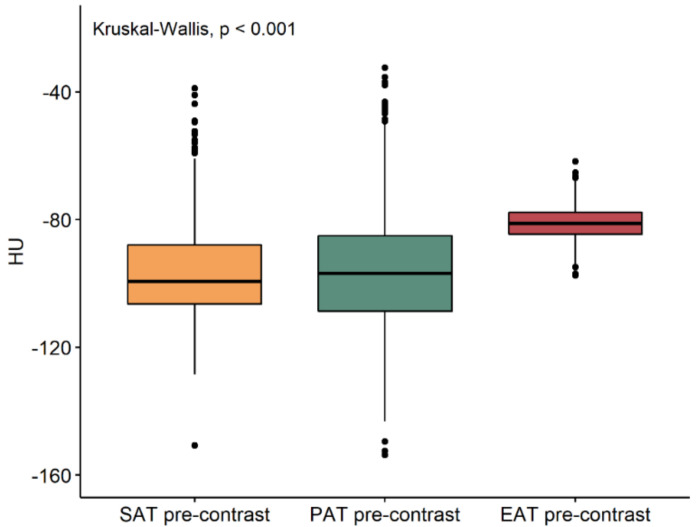
Boxplot of subcutaneous (SAT), paracardiac (PAT), and epicardial (EAT) adipose tissue unenhanced density values. EAT density is significantly greater than SAT and PAT.

**Figure 4:**
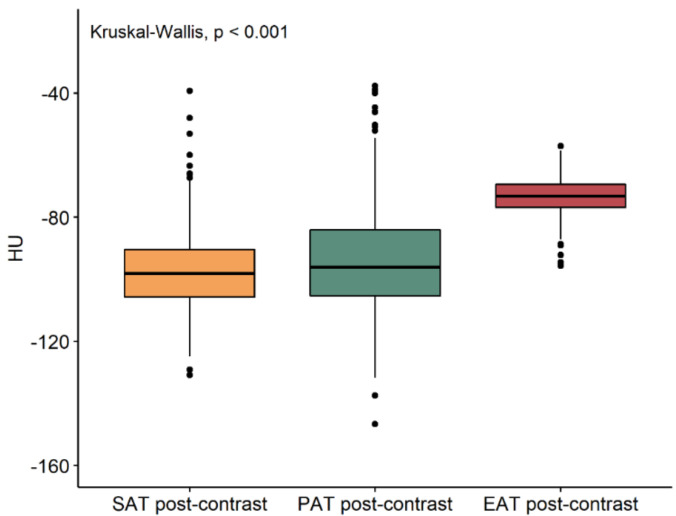
Boxplot of subcutaneous (SAT), paracardiac (PAT), and epicardial (EAT) adipose tissue post-contrast density values. EAT density is significantly greater than SAT and PAT.

**Figure 5:**
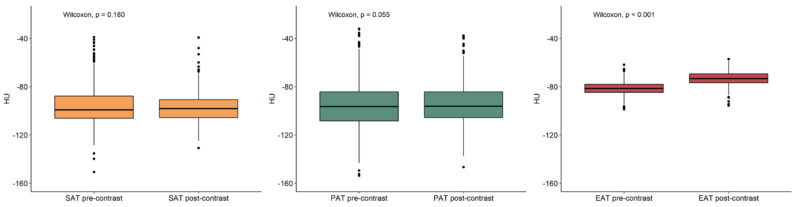
Comparison of subcutaneous (SAT), paracardiac (PAT), and epicardial (EAT) adipose tissue pre- and post-contrast values. EAT density values significantly increased after contrast administration.

The median density values of subcutaneous, paracardiac, and epicardial adipose tissue on unenhanced and contrast-enhanced scans are reported in Table 1. On enhanced scans (Figure 3), there was no difference in subcutaneous and paracardiac adipose tissue density values (*p* = 0.380), while epicardial adipose tissue density values were significantly higher than those of subcutaneous and paracardiac adipose tissue (*p* < 0.001). On contrast-enhanced scans (Figure 4), a difference was found between subcutaneous and paracardiac adipose tissue, the latter being slightly denser (*p* = 0.002), with epicardial adipose tissue still having higher density values than subcutaneous and paracardiac adipose tissue (*p* < 0.001). Furthermore, epicardial adipose tissue density significantly increased after contrast administration (*p* < 0.001), with a median difference of 8.2 HU (IQR 4.3–12.1 HU), whereas either a borderline significant or no significant difference was observed between unenhanced and contrast-enhanced density values of paracardiac and subcutaneous adipose tissue (*p* = 0.055 and *p* = 0.180 respectively), as shown in Figure 5. 

Concerning CAD, no differences were found among subcutaneous, paracardiac, and epicardial adipose tissue density values between CAD and non-CAD patients, regardless of contrast administration (Table 2). 

## 4. Discussion

The role of adipose tissue CT density as a biomarker of cardiovascular pathology has gained widespread interest over the last few years [22]. In particular, epicardial adipose tissue density, given the peculiar nature of this particular adipose tissue, has been the object of a number of studies which have yielded contrasting results, leaving its potential as a biomarker for CAD still not entirely clarified.

The density values found for epicardial adipose tissue in this work, significantly higher than those of paracardiac and subcutaneous adipose tissue (*p* < 0.001 for both) fit well with the notion that the anatomy and physiology of epicardial adipose tissue are different than those of paracardiac and subcutaneous adipose tissue. In fact, higher values of fat density may reflect the beige nature of epicardial adipose tissue and its increased metabolic activity, as opposed to paracardiac and subcutaneous adipose tissue, which are mostly white [23]. More so, while the embryological origin of epicardial adipose tissue lies in the splanchnopleure of the mesoderm, paracardiac adipose tissue originates from the thoracic mesenchyma [24]. Thus, these different adipose tissues prove diverse both in developmental origin and nature. To further back these notions, epicardial adipose tissue density appears to increase on contrast-enhanced scans compared to unenhanced scans, favoring the hypothesis that it may present with a more developed vascularization to support its metabolic activity. This result is in line with findings from one study by La Grutta et al. [12], who observed that epicardial adipose tissue density was significantly higher on contrast-enhanced CT scans. However, these authors reported density values lower than ours on unenhanced scans and higher values on contrast-enhanced scans, such differences being possibly due to different methods of epicardial adipose tissue segmentation. In fact, the authors of this work utilized a semiautomatic segmentation method which included the whole pericardium, whereas we manually segmented epicardial adipose tissue on one representative slice.

We also observed borderline significantly higher paracardiac adipose tissue density values on contrast-enhanced scans compared to unenhanced scans (*p* = 0.055). This could most likely be due to paracardiac adipose tissue being composed by a combination of white and beige adipose tissue, thus preserving a higher metabolic activity compared to subcutaneous adipose tissue, albeit lower than that of epicardial adipose tissue [13,15].

Concerning CAD, we did not observe differences in epicardial adipose tissue density values between patients who had a CAD-negative cardiac CT and those who had a CAD-positive CT, defined as having at least either two mild or one moderate coronary stenoses. In this regard, findings from previous works have been contrasting, with studies suggesting that epicardial adipose tissue density may either increase or decrease in patients with CAD. For instance, Lu et al. [25] observed lower epicardial adipose tissue density values on unenhanced CT scans in patients presenting with high-risk plaques. Similarly, Goeller et al. [26] reported lower epicardial adipose tissue density values on unenhanced scans in patients with a higher incidence of major cardiac events, and Franssens et al. [22] noted that a lower epicardial adipose tissue density on contrast-enhanced CT correlated with higher calcium scoring values. Conversely, Pracon et al. [27] found higher epicardial adipose tissue density values on contrast-enhanced scans in CAD patients compared to patients without CAD, while Mahabadi et al. [9] reported higher epicardial adipose tissue density values on unenhanced scans for patients with type 1 myocardial infarction, i.e., caused by an acute atherothrombotic coronary event. In addition, one further study by Nerlekar et al. [19] indicated that epicardial adipose tissue density tends to decrease with increasing age, which is also usually associated with the progression of cardiovascular risk factors. 

Of note is that a lower density of adipose tissue could be due to an increase in free fatty acids accompanied by an overall volumetric expansion, and different works have described an inverse correlation between epicardial adipose tissue density and volume [10,23,25]. On the other hand, increases in its density could arise from inflammation and fibrosis, two processes that are tightly related to CAD. Indeed, larger epicardial adipose tissue volumes have been linked to the decrease of cytokines (which are protective against inflammation) and a consequent increase in tumor necrosis factor α (a powerful inductor of inflammation) [28]. Hence, the reported contrasting findings and the lack of differences in density values between patients with and without CAD in our population could be explained by different mechanisms that occur in epicardial adipose tissue with the development of CAD. In fact, we might hypothesize that in patients exhibiting decreased epicardial adipose tissue density CAD might be more correlated with a gradual increase in free fatty acids typical of metabolic syndrome [29]. Conversely, in patients exhibiting increased epicardial adipose tissue density, more pronounced perivascular inflammation and fibrosis might prevail. This hypothesis could be navigated further by assessing subgroups in large populations, including clinical findings and metabolic biomarkers in the analysis. Additionally, perhaps tools enabling radiomic analysis could also prove useful in this setting, as they might help analyze image patterns in epicardial adipose tissue density which are not visible to the human eye [30].

Our work presents some limitations. First, our segmentations for epicardial, paracardiac, and subcutaneous adipose tissue only related to limited areas, as epicardial adipose tissue was only segmented on one individual slice, and density values for paracardiac and subcutaneous adipose tissue related to individual ROIs with a 1-cm diameter. Nevertheless, we aimed to position the ROIs in areas that could be well-representative of each respective adipose tissue, and the density values we observed for epicardial, paracardiac, and subcutaneous adipose tissue agree with previous works [15,23]. Second, the sample size of our work was calculated focusing on epicardial adipose tissue; therefore, we may not definitively affirm that paracardiac adipose tissue density does or does not vary between unenhanced and contrast-enhanced scans, given the borderline significant *p*-value (*p* = 0.055) pertaining to that difference. Third, our non-CAD patients were still individuals who had been referred for a cardiac CT; therefore, they may not be well representative of a healthy population. Nevertheless, the population we analyzed was a sample of usual clinical targets for cardiac CT, a pool where epicardial adipose tissue as a biomarker could be considered of interest. 

In conclusion, while epicardial adipose tissue shows density values higher than those of subcutaneous and paracardiac adipose tissue, in agreement with a more pronounced epicardial adipose tissue metabolic activity, we did not find any trend towards a decrease or an increase in epicardial adipose tissue density in the presence of CAD. Further studies may be warranted to assess whether relevant tendencies could be observed in particular subgroups, or if there could be some common features in epicardial adipose tissue density in CAD patients compared to non-CAD patients or healthy subjects.

## Figures and Tables

**Table 1:** Fat tissues density values.

	Unenhanced Scans	Contrast-Enhanced Scans	*p*_pre-post_
SAT (HU)	−99.1 (−106.2 to −87.6)	−98.1 (−105.8 to −90.6)	0.180
PAT (HU)	−96.4 (−108.4 to −84.1)	−96.1 (−105.3 to −84.0)	0.055
EAT (HU)	−81.5 (−84.9 to −78.0)	−73.4 (−76.9 to −69.4)	<0.001 *

SAT, subcutaneous adipose tissue; PAT, paracardiac adipose tissue; EAT, epicardial adipose tissue. * Denotes statistical significance.

**Table 2:** Comparison between fat density values among coronary artery disease (CAD) and non-CAD patients.

	No CAD	CAD	*p*
SAT			
SAT pre (HU)	−99.4 (−105.9 to −87.5)	−97.5 (−106.6 to −88.0)	0.739
SAT post (HU)	−99.0 (−106.6 to −90.8)	−96.9 (−103.7 to −90.4)	0.092
PAT			
PAT pre (HU)	−96.5 (−108.3 to −82.9)	−96.1 (−108.2 to −86.9)	0.484
PAT post (HU)	−95.9 (−104.9 to −82.4)	−96.5 (−105.7 to −85.2)	0.326
EAT			
EAT pre (HU)	−81.2 (−84.6 to −78.0)	−81.8 (−85.3 to −77.8)	0.408
EAT post (HU)	−72.6 (−76.9 to −69.2)	−74.0 (−77.0 to −70.0)	0.232

SAT, subcutaneous adipose tissue; PAT, paracardiac adipose tissue; EAT, epicardial adipose tissue; Pre, non-contrast; post, post-contrast.

## Data Availability

The data presented in this study are available on request from the corresponding author. The data are not publicly available due to privacy concerns.
